# Address

**Published:** 1872-07

**Authors:** E. J. Waye


### ADDRESS

### Upon Retiring From the Presidency of the Northern Ohio Dental Society.

BY DR. E. J. WAYE.

*Mr. President and Gentlemen of the Association:*

The speech of the retiring President is like saying grace after meat; it is as though, at a rich banquet, wherein all that could delight the eye or tempt the appetite, treasures of culinary art—the united offerings of artists skilled in their respective departments left no desire ungratified, and all were filled to repletion, the host were to be called upon to furnish for appetites already satiated a dish of his own preparation, a dish which should quicken palates already cloyed by enjoyment, and lend a new jest to satiety.

Add to the perplexity of his situation the fact that, before accepting his attempts at pleasing, he is first stripped of whatever of dignity or authority may have been conferred by his position, leaving him to his own *unaided* resources, and you may form some conception of the embarrassments of his situation.

At such a feast, gentlemen, you have been the favored guests. Yours has been the rich, substantial, and nutritious meat of wisdom, learning and experience, seasoned with the attic salt of wit, decorated with the flowers of rhetoric and fancy, and pledged in the mellow old wines of true courtesy and professional regard.

If the extraneous dish shall be found less toothsome and exhilarating, the circumstances under which it is concocted may, it is hoped, furnish in part, at least, its apology.

The profession, in the interest of which we are again assembled on this beautiful island, is now honored as amoug the first in importance of the specialties of medicine and surgery; and in its benign ministrations to suffering humanity ranks second to none; numbering among its sons many of the finest minds, and most earnest and indefatigable workers, in the fields of scientific research and investigation, and among its institutions colleges whose curriculum, in extent and thoroughness, are not surpassed by those for the study of general medicine and surgery. It has also societies, associations, libraries, journals, and a literature. As a profession it is markedly progressive, with tendencies toward culture and a high standard of professional excellence. Its beneficent ministrations are so well known and recognized that wise legislators do not hesitate to frame laws and enactments making it a criminal offence to practice under its name without first undergoing a searching examination by a board of dentists chosen from the State Dental Socicty for that purpose, or the possession of a diploma from some dental college of standing and respectability.

The dentist of to-day, if he be a man of education and professional ability (and the time is rapidly approaching when none but such will be permitted to enter its practice), readily takes a fair position in society, commands the patronage of the most refined and cultivated persons in the community; his skill is recognized, his services sought and appreciated, and a fee worthy of such ability and skill, faithfully rendered, is ungrudgingly bestowed. Such is the proud position of dentistry and dentists to-day. Now, gentlemen, how long is it since our profession arrived at such an enviable position? What have been the causes which led to its present recognition and appreciation?

It was but yesterday that dentistry was considered a trade rather than a profession, and dentists were regarded by medical men as mere "*tooth carpenters*," men who, having some mechanical skill and ingenuity, might insert a sorry substitute for a lost front tooth, or plaster up a decaying crown with amalgam. But as for any true knowledge of the organ, which they so ruthlessly scraped, filed, plastered, or wrenched out, did the medical profession or any one else expect they knew aught? or that any such knowledge was necessary or desirable?

How long is it, indeed, since the name of dentist signified simply an itinerating perambulator, who, with separating files, a few ill-shaped excavators, a quarter or a half silver dollar

(as his finances warranted), and a vial of quicksilver meandered about the country persuading the people that "dame nature," was no dentist as compared with *him*. For had not she placed the teeth in such close proximity that decay must occur? and that separation with the file was the potent and only remedy; which, while curing decayed teeth and protecting the sound from danger, insured also that nice and equal separation, so essential to true beauty and symmetry, as understood by the fashionable dentists of that day.

The fact that the barber united with his vocation two professions, "surgeon and dentist," either of which is now thought sufficient to tax the full powers and capacity of any one man, however gifted, is in itself a striking proof of the limited field of knowledge and practice *at that time*, as well as the wonderful advance in both professions in our own. And this brings me to the consideration of a few of the many causes which brought us to our present status.

The list, arranged according to our own ideas, would read something like this:

1. The adoption of the dental profession by men of genius, possessing a medical education.

2. As the result of this, the institution of dental colleges,

3d. The discovery of the adhesive property in gold, and the improved instruments and modes of operating consequent thereupon.

4. The spread of intelligence among dentists, through its journals and literature.

Last—though by no means least—"Association" and its fruits, viz: free interchange of thought; modes of operating; clinics; codes of ethics; protective enactments, etc., etc.

A passing notice only is attempted, lest we weary your patience beyond endurence.

While we would by no means under-estimate the merits of those ingenious minds whose skill in the infancy of the profession devised the many new and improved methods for the insertion of artificial teeth, we must still think that to the few who, to mechanical genius of a high order added the knowledge obtained at the schools of medicine, and the spirit of inquiry, the habit of research, and experiment therein encouraged and inculcated, which led them out of the merely mechanical and artificial into the study of the organs themselves; their structure, and the diseases which caused their premature decay and loss, we must look as the founders of the dental colleges.

While the mere mechanic was in many instances the ingenious inventor of new modes and instruments, he was nevertheless reticent, jealous of his brethren, and resolved that none should share with him in improvements of his own devising. Such are not the men who found colleges, or bestow the skill so industriously acquired upon their more ignorant brethren.

And it is not to that class we should naturally look as the founders of any institutions for the benefit of dentistry as a profession.

But the student of medicine was a different man; his studies had not been pursued in solitude. He had been surrounded by young men in pursuit of the same knowledge as himself, and beset with the same difficulties.

Thrown together in the lecture room, they listened to the same teachings, or witnessed in the clinic and dissecting room similar operations and demonstrations.

A strong chain of sympathy and friendship bound them together, ending not with their college honors, but felt and enjoyed through after life.

Such a man adopting a new profession naturally felt a strong desire for like sympathy within it, instituted at once a search, and if by good fortune one sympathizing brother was found, ten to one but he knew another of like sympathies, and he a third, and so, feeling the good of that brotherly and professional regard which they had so enjoyed in the past they proposed to found for the good of the coming man a dental college, wherein might be acquired that knowledge which is so hard to obtain outside its walls, and at the same time cultivate those brotherly sympathies so dear to every man in every profession.

To the dental college we owe in great part that higher and nobler branch of the profession, that particular speciality which cares for the natural organs; the practice of which involves a thorough knowledge of the anatomy, physiology; and pathology of the teeth, their structure and constituents, also what tends to their injury or disease, and the local and constitutional treatment necessary to their restoration to health.

Not this alone, but the structure and diseases of all the contigous parts as well. Over and above all this, the operative dentist must possess that nice and accurate mechanical skill which shall enable him to stop, not merely a simple cavity easy of access, but to restore the broken down parts with gold, preserve the contour, and finish of the natural organ, and unite the gold and the tooth by a joint as impervious to air or fluid as either the gold or enamel.

This knowledge and this art may be and is acquired in the dental college.

The discovery of the adhesive property in gold marked an era in the history of dentistry. To that discovery we are in a great measure indebted for the strictly operative branch in our profession. Previous to that time, it is true, gold fillings had been made by some few pains-taking and skillful men, which doubtless arrested decay and preserved the tooth. But the principle on which they relied for success, while in favorable localities and where the walls of the cavity were of sufficient strength, namely, packing the gold with flat-sided instruments against the sides of the cavity, and retaining it with a wedge in the center; though successful in small cavities with thick and strong walls, failed entirely in that much larger class, whose thin sides, frail and disintegrating, could not withstand the necessary pressure, or whose shallowness would not permit of their being properly shaped without danger of exposing the pulp.

Contour filling, the crown of operative dentistry now, was out of the question, and impossible without adhesive gold; and the best that the most skillful hoped to accomplish (and more than most succeeded in doing), was to fill the cavity so full as to permit of a tolerable finish, flush with its edges. Up to the introduction of adhesive gold, and indeed for some time after, filling teeth was believed by most to be of little use; and so far as my own experience goes, it was of little use—not one in five of the fillings I saw then (and I suppose they were as good as the average) subserving any useful purpose whatever, save to retain upon their rough and unpolished surfaces, or within their interstices, the various impurities which usually induce decay. So strongly was this felt that the best men preferred to devote their attention almost exclusively to artificial work. Gold being then the material generally worn their production required a greater degree of skill, and also it may be because that branch of the profession was at that time the most remunerative. What adhesive gold, improved instruments, and that indispensable adjunct the rubber dam, are now doing, none present need be told, for all have beheld the marvels of art, beauty and usefulness, wrought out by hundreds of skilled dentists throughout the land—marvels which only the discovery of some new material, of the color of the teeth, and the indestructibility of gold, shall enable us to give color as well as contour.

Of the originators of dental associations no extended account will be attempted; their names are familiar to most of us, and will not be mentioned here; nor can the benefits which their generous and noble efforts have conferred upon our profession be sketched at this time, save in the most brief and unsatisfactory manner. But enough may be elicited to establish their claims to our most heart-felt emotions of gratitude and regard.

Before associations had brought men into contact, taught them to compare methods, interchange ideas, exchange courtesies, and infused into them a noble emulation in acquiring, and a generous and manly delight in imparting whatever of knowledge or skill they possessed, dentistry as a profession scarcely existed. "Every man for himself" was the motto. Isolation from his brother dentist, jealousy of another's success, a miserly guarding of every new method or material, lest his brother by its acquisition should reap the benefits which he claimed as his own; a hatred and contempt of educated dentists, an over weening conceit for his own wonderful secrets and skill, and a corresponding depreciation of every one else—these were the strongly marked characteristics of the dentist before the dawn of the "Association."

The presence of so goodly a number of the profession today, from various parts of the State (and I see some from a sister State), assembled for mutual benefit, and the kindly interchange of thought and courtesy, is the best possible evidence that a different feeling now animates the profession. A desire to do something for the general good, an earnest and heart-felt interest in the education, culture, and elevation of its present members only, but of the coming men also, is the governing impulse now.

It is "Association" which has wrought this change. It has substituted for ignorance knowledge for intolerance and conceit, liberality and manly courtesy. It has given to the profession new methods and new inventions. To it we are largely indebted for dental colleges, and entirely for protective enactments.

To "Association," beyond all other causes combined, do we owe our present prosperity and our future elevation. When the association, which provides already the Examining Board, shall also own and control the colleges which grant diplomas, the profession will have reached that higher position so much to be desired, and community will be protected from the empiric and the charlatan. If this be true, it should stimulate us to renewed earnestness in the support of our various societies. Their influence for good upon the profession at large is incalculable while to the individual they are an indispensable stimulant to that growth and development which marks the dentist of to-day.

